# Supplementary material for: Fundamental aspects to localize self-catalyzed III-V nanowires on silicon
Source: Nat Commun. 2019 Feb 20;10:869. doi: 10.1038/s41467-019-08807-9 (PMC6382777; doi:10.1038/s41467-019-08807-9)
Supplement: Supplementary file 1 — Supplementary Information [file 41467_2019_8807_MOESM1_ESM.pdf]

Fundamental aspects to localize self-catalyzed III-V nanowires on silicon

J. Vukajlovic-Plestina[1], W. Kim[1], L. Ghisalberti[1,2], G. Varnavides[2], G. Tütüncuoglu[1], H. Potts[1], M. Friedl[1], L. Güniat[1], W. C. Carter[1,2], V. G. Dubrovskii[3], A. Fontcuberta i Morral[1,4]
[1] Laboratory of Semiconductor Materials, Institute of Materials, EPFL, 1015 Lausanne, Switzerland
[2] Departments of Materials Science and Engineering, MIT
[3] ITMO University, Kronverkskiy prospekt 49, 197101 St. Petersburg, Russia
[4] Institute of Physics, EPFL, 1015 Lausanne, Switzerland

Supplementary notes

Supplementary Note 1. Experimental details

Substrate design and preparation

For the growth experiments, we used (111) Si substrates with a thin layer of thermally grown silicon oxide that was patterned by arrays of holes with different diameters. The nominal diameters of the holes were from 30 to 90 nm with the increment of 15 nm (Suppl. Fig. 1.), while different aspect ratios were achieved by using different oxide thicknesses: 10 nm, 15 nm and 20 nm. Each substrate had arrays with different inter-hole distances (pitches), but we found that yield does not depend on this parameter. Therefore, we performed the analysis on the arrays with 400 nm pitch. Patterning process was realized by electron beam lithography followed by dry (reactive ion etching – RIE) and wet etching (buffered hydrofluoric acid - BHF). Combining these two etching steps was necessary to obtain good and reproducible profile of the holes. The last step in the substrate preparation before growth was so called "last dip". It refers to the 2 s dip in the BHF bath to remove the native oxide created at the bottom of the opening. The diameters of the holes as well as the oxide thickness can differ from the nominal values due to the etching processes, especially due to the "last dip". For this reason, substrates were thoroughly studied by atomic force microscopy (AFM) and spectroscopic ellipsometry (SE). AFM was used to precisely determine the diameter of the holes and to check their profile, while SE was used to measure the exact oxide thickness (a summary of these results obtained for one batch of samples is presented in Suppl. Fig. 2.).

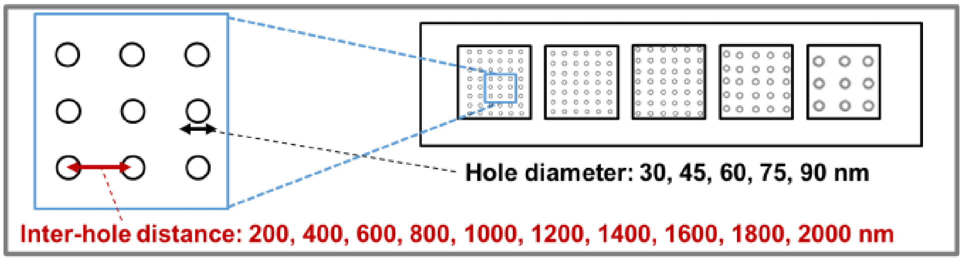

Supplementary Figure 1: *Substrate design.*

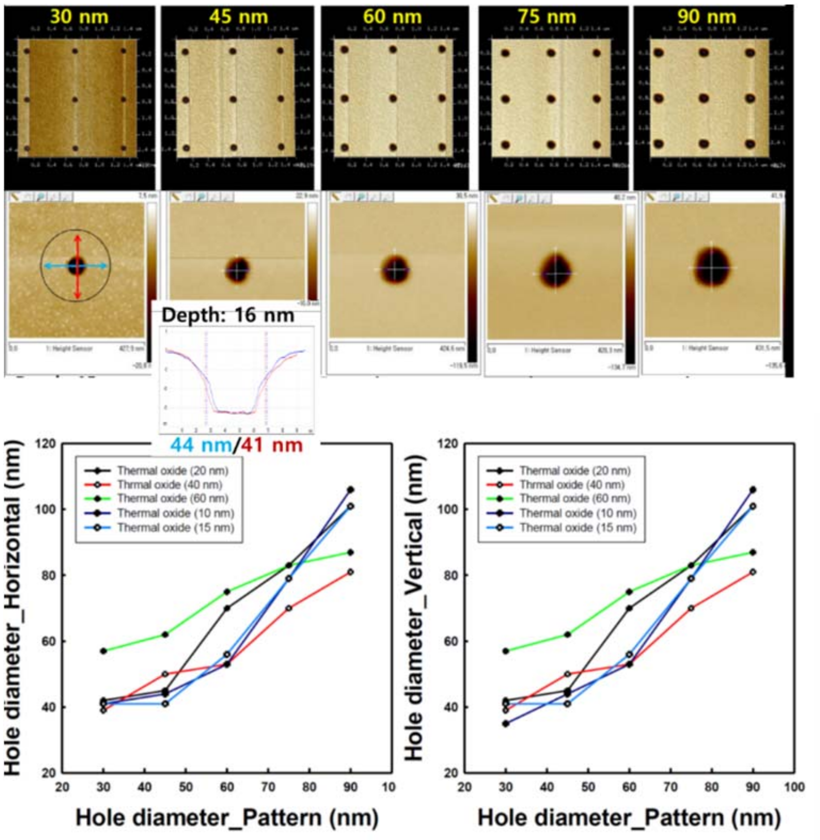

Supplementary Figure 2: *Substrate characterization. AMF is used to complete the ellipsometry measurements of the oxide thickness, determine the hole diameter and verify that the holes are well defined. Results shown here are obtained for one batch of samples.*

Growth conditions

All our growth experiments were performed using the same material fluxes and growth temperature: a gallium flux corresponding to the GaAs growth rate of 1 Å/s, an As_4 partial pressure of 2×10^{-6} Torr and a temperature of 635°C. The gallium pre-deposition time and the growth time were varied.

Supplementary Note 2. Aspect ratio of the holes influencing the yield

As presented in the main text, we found that the optimal aspect ratios (d/h) of the holes is between 4 and 6, which is consistent with the work of Plissard[1] et al. Here, Suppl. Fig. 3. shows the overview of the arrays with different aspect ratios, obtained by varying the oxide thickness (10, 15 and 20 nm) and hole sizes (30, 45, 60, 75, and 90 nm nominally; the real hole sizes are slightly bigger due to the last BHF dip). One can see how the best yields shift toward larger

holes with increasing the oxide thickness, which demonstrates a strong correlation between the aspect ratio of the hole and the yield.

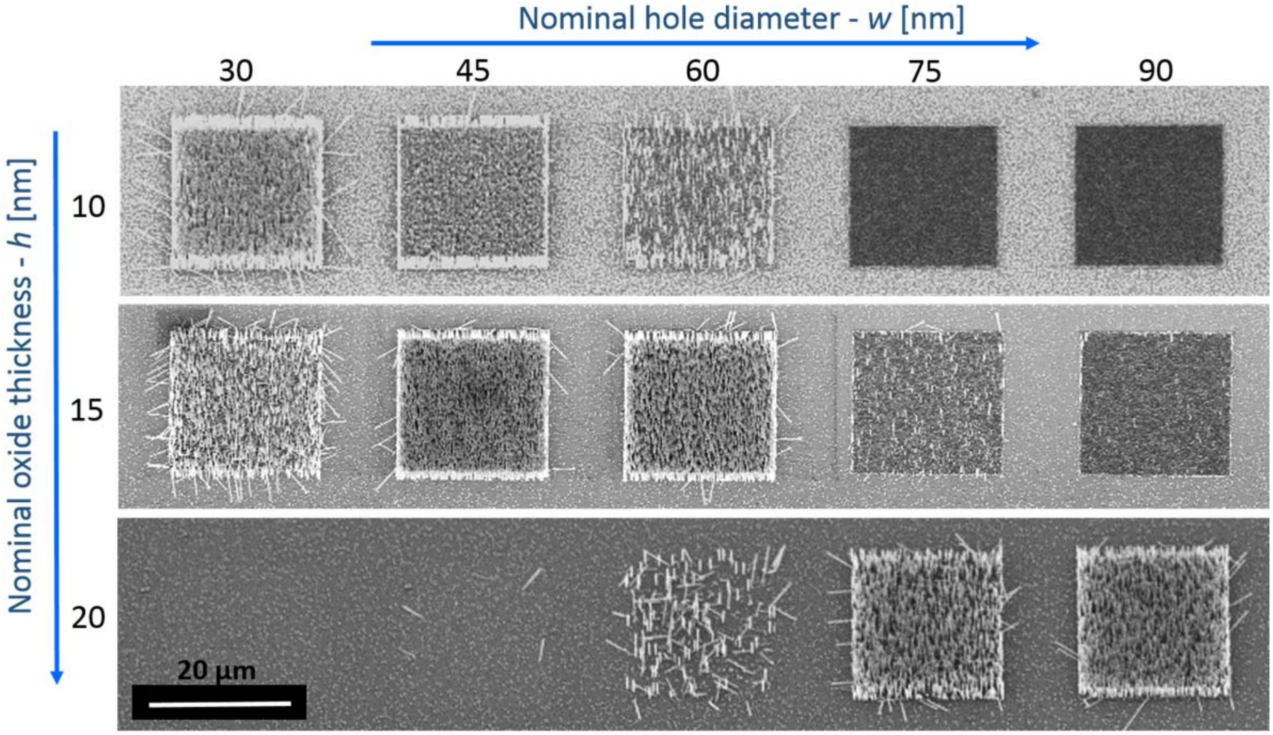

Supplementary Figure 3: *20° tilted SEM images of the arrays with different oxide thicknesses and hole diameters, demonstrating the correlation between the hole aspect ratio and the yield.*

Supplementary Note 3: Droplet – hole configuration and its impact on the yield

Besides the aspect ratio, we found that the droplet configuration within the openings also influences the yield. As explained in the main text, we found that only droplets symmetrically filling the hole give rise to the subsequent vertical growth of nanowires. This can clearly be seen in Suppl. Fig. 4. Suppl. Fig.4 a. shows the AFM images of the droplets within the holes of different sizes (30, 60 and 90 nm), formed after different gallium pre-deposition times (5, 10 and 15 minutes). One can observe how the droplets start at the edge, continue to grow and finally fill the hole. Suppl. Fig. 4 b. shows the comparison of the yields in differently sized holes for different gallium pre-deposition times. Clearly, the best yields shift toward larger holes with increasing the gallium pre-deposition times. The same results are presented in the Suppl. Fig. 5, in a more quantitative way, by showing the statistics and zoomed-in SEM images of some NW arrays. The improvement of the yield in 75 nm holes from almost 0 % for 5 and 10 minutes of

gallium pre-deposition to 50 % for 15 minutes of gallium pre-deposition clearly demonstrates the importance of fully filling the holes with gallium.

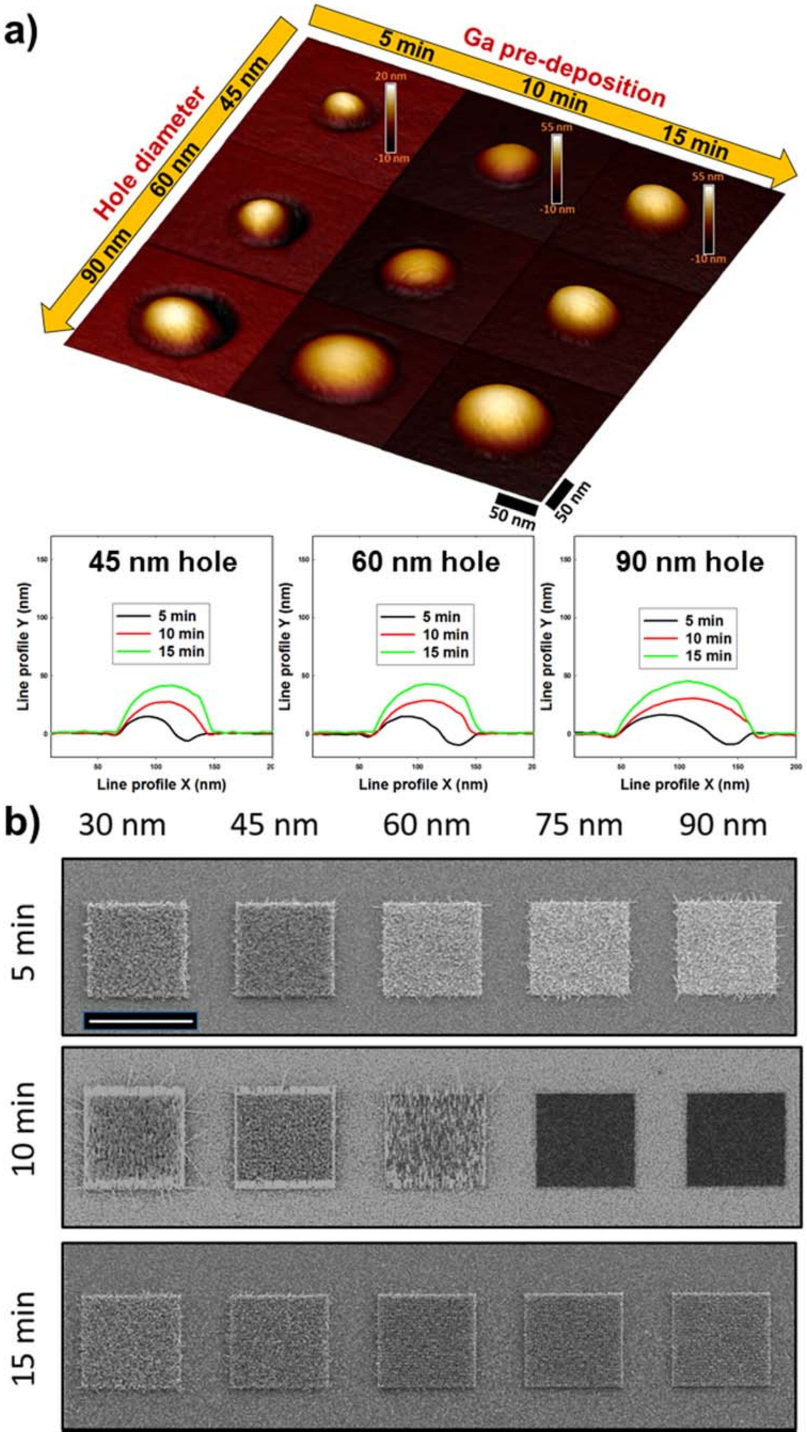

Supplementary Figure 4: *(a) AFM images of the gallium droplets within the holes formed after different gallium pre-deposition times and their line profiles. (b) The overview of the arrays with different hole sizes and gallium pre-deposition times; the scale bar is 20 µm.*

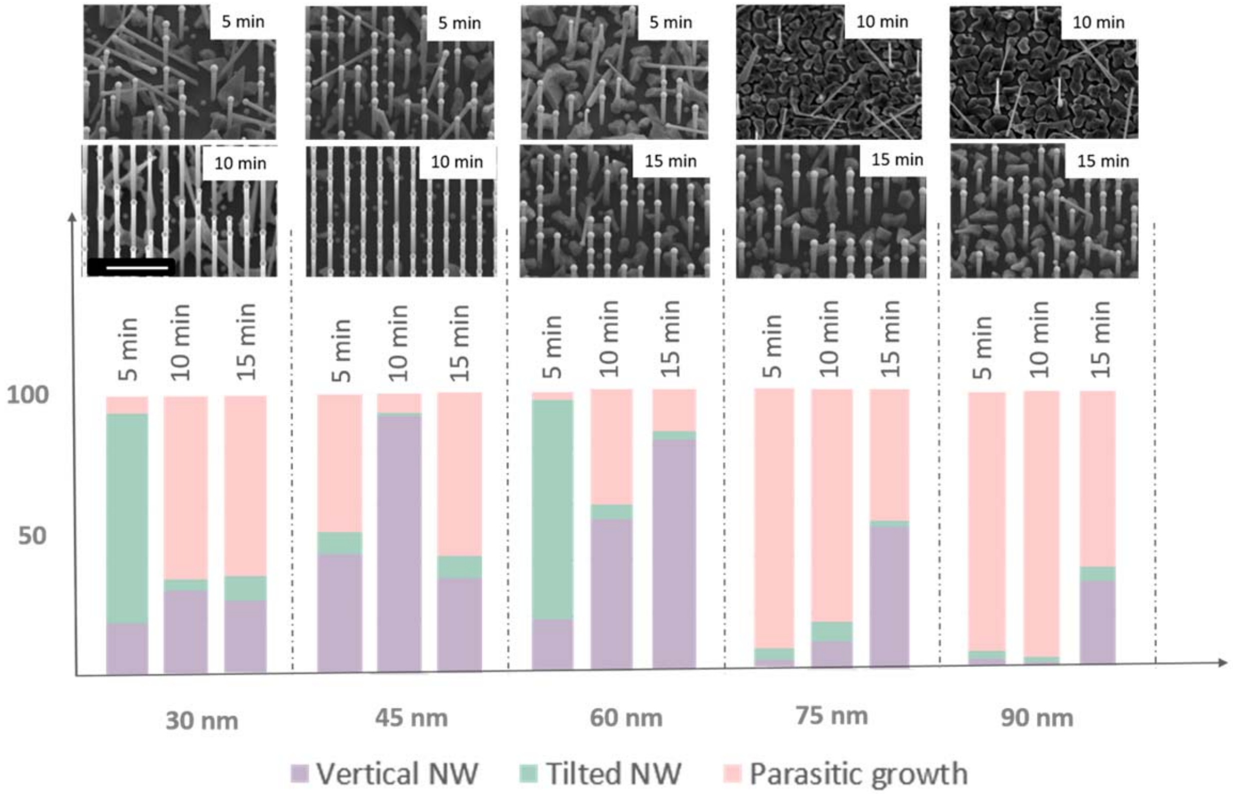

Supplementary Figure 5: *The yield statistics in the arrays with different hole sizes and gallium pre-deposition times. Violet parts of the bars correspond to the yield of vertical nanowires. The gallium pre-deposition time is indicated for each bar, with the representative SEM images shown on top. The scale bar on the SEM images is 2 µm and the tilt angle is 20˚.*

Supplementary Note 4: Equilibrium shape of gallium droplets in cylindrical holes

The figures shown in the main text represent the computed shapes corresponding to the minimum surface energy of a liquid droplet wetting cylindrical hole as a function of the droplet volume. These results are based on the simulations of the wetting behavior of constrained sessile droplets performed using Surface Evolver, a finite element based software.

The simulations were performed as follows. First, we identified 5 main pinning sites of the droplets, as shown in the insets to Suppl. Fig. 6. For each configuration, the calculations toward the equilibrium state were performed at a fixed droplet volume and contact angles. The variations of the normalized energies of the liquid as a function of its volume for different configurations are shown in the main panel of Suppl. Fig. 6.

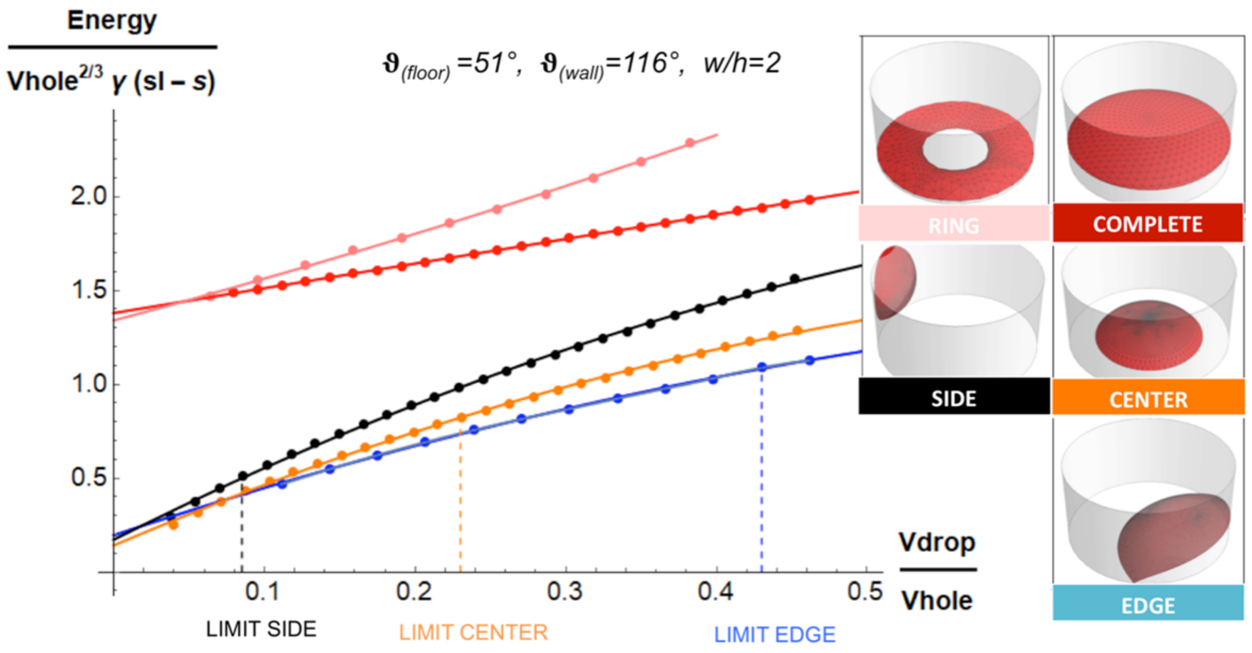

Supplementary Figure 6: *Normalized energies of the droplet versus its volume for five different configurations illustrated in the insert: ring-like (pink line), complete wetting (red line), at the side (black line), in the center (orange line) and at the edge (blue line) of the hole.*

Using the interfacial energies estimated from the experimentally measured contact angles, it was possible to define the wetting morphology of the system considered. The values used are: 51° for the contact angle at the bottom of the hole (corresponding to wetting of the bare silicon with gallium), and 116° at the walls (representing the unfavorable wetting of the oxide sidewalls).

Starting from each predefined initial shape, Surface Evolver computes the position of vertexes according to the forces acting upon them in order to minimize the surface energy of the system for a given volume. The iteration methods used in our calculations are the gradient descent method and the conjugate gradient method. By setting a convergence criterion for the energy difference between the consecutive iterations, we find the equilibrium shapes of the droplet for each shape and compare their energies to determine the preferred configuration.

Based on these calculations, we conclude that at very small volumes, the droplet prefers to wet the nanohole center, avoiding the sidewalls. As the volume increases, the preferential droplet position is at the edge of the hole, but such configuration is geometrically limited by the aspect ratio of the hole itself. Therefore, further increasing the volume leads to the complete wetting configuration, consistent with our experimental results.

Supplementary Note 5: Shapes of GaAs crystals forming in the holes

After removing the gallium droplets, we found unexpected geometries of the remaining GaAs crystals, such as step-like or ring-like (donut) geometry. By performing a detailed AMF analysis, we determined that heights of these structures are much larger than monoatomic, as demonstrated in the Suppl. Fig. 7.

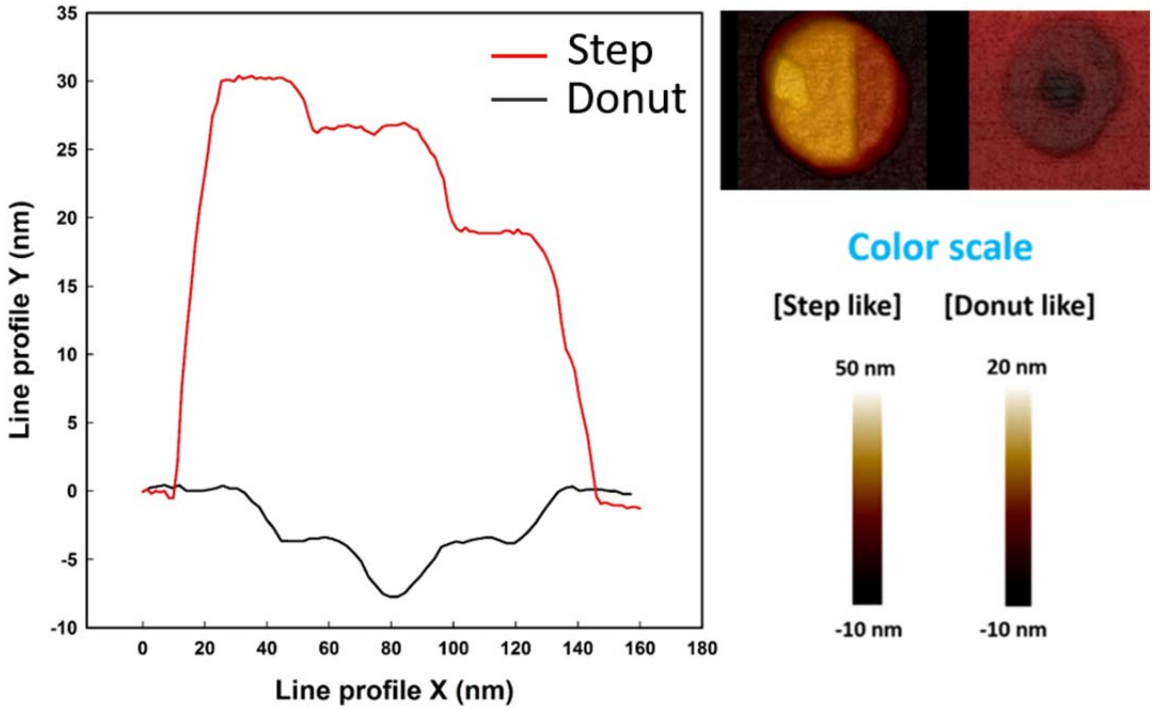

Supplementary Figure 7: *AFM images and the corresponding line profiles of different GaAs crystals at the bottom of the holes, showing their heights that appear much larger than monoatomic.*

Supplementary Note 6: Modeling of GaAs nucleation in SiO_2/Si(111) templates

In this modeling, we try to understand the unusual geometry of GaAs underneath gallium droplets in the holes, with either stepwise or ring geometry at the outer periphery of the holes and restricted by the steps that are much higher than monoatomic. In the standard VLS growth of developed nanowires far away from the substrate, theoretical considerations [1-3] and *in situ* growth monitoring [4,5] reveal mononuclear formation of planar nanowire monolayers which proceeds layer-by-layer so that the flowing steps are always monoatomic. However, the VLS growth within the holes is different – first, GaAs crystal nucleates on the lattice mismatched

silicon substrate and, second, the crystal has lateral solid-solid interface with the SiO_x mask rather than free sidewalls in contact with vapor.

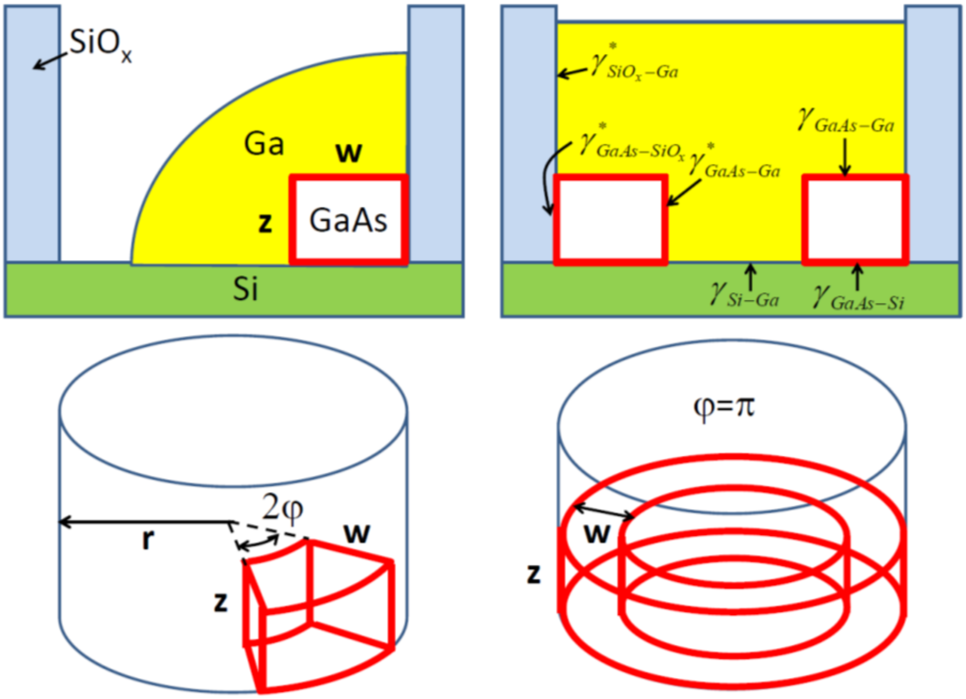

Supplementary Figure 8: *Schematics of the geometry of GaAs (in red) growing from liquid gallium in a SiO_x/Si(111) hole. The hole walls are SiO_x and the bottom is silicon. The opening angle φ can vary from 0 to π depending on the gallium droplet and seed size. The GaAs crystal is a section of cylindrical ring of height h and width w, with the aspect ratio w/h.*

Initially, the gallium droplet may occupy a part of the hole (for smaller gallium volumes) or completely fill the hole (for larger gallium volumes) as shown schematically in Suppl. Fig. 8. In the former case, the gallium droplet should be positioned at the edge of the hole for surface energetic reasons [6,7]. The initial droplet volume can be characterized by the angle φ showing which portion of the hole bottom is covered with liquid gallium ($\varphi < \pi$ for incomplete and $\varphi = \pi$ for complete filling). Assuming cylindrical geometry, we consider free energy of forming a GaAs crystal at the outer periphery of the hole, with the opening angle 2φ ($\varphi < \pi$ corresponds to incomplete and $\varphi = \pi$ to complete ring), width w and height z [see Suppl. Fig. 7.], at a fixed volume of GaAs. The latter is given by $V = zS$, where $S = \varphi r^2 - \varphi(r-w)^2 = \varphi r^2 x(2-x)$ is the surface area of the base and $x = w/r$ is the width of the crystal divided to the hole radius $r = d/2$ (the case $x \to 0$ corresponds to planar growth on the inner sidewalls of SiO_x and $x = 1$ to planar growth on the silicon bottom). Disregarding the volume term with chemical potential (which is the same for any configuration of the GaAs crystal at a fixed V), we can write

$$\Delta G = \frac{\lambda \varepsilon_0^2 V}{1 + Az/w} + 2\varphi r z(\gamma^*_{GaAs-SiO_x} - \gamma^*_{SiO_x-Ga}) + 2wz\gamma^*_{GaAs-Ga} + 2\varphi(r-w)z\gamma^*_{GaAs-Ga} + S(\gamma_{GaAs-Si} + \gamma_{GaAs-Ga} - \gamma_{Si-Ga}). \quad (1)$$

Here, the first term describes the elastic energy contribution induced by the lattice mismatch between GaAs and Si [8-11], with $\lambda = 1.4 \times 10^{11}$ J/m^3 as the effective elastic modulus of bulk GaAs, $\varepsilon_0 = 0.04$ at the lattice mismatch, and $A \cong 7.5$ as the relaxation coefficient [10]. Very importantly, coherent growth of GaAs on silicon requires the radius of the GaAs crystal to be smaller than the critical radius of 53 nm [11], which is fulfilled in our case and hence all GaAs NWs should be free of dislocations at the base. The next three terms stand for the surface energies of vertical sidewalls of GaAs, with γ^* representing the surface energies of the corresponding vertical interfaces as shown in Suppl. Fig. 7 (the GaAs-SiO_x and GaAs-Ga surfaces are created and the SiO_x-Ga one is eliminated upon nucleation). The last term stands for the in-plane surface energy change, with γ representing the surface energies of the corresponding in-plane interfaces (the GaAs-Si and GaAs-Ga surfaces are created and the Si-Ga one is eliminated upon nucleation).

Introducing dimensionless free energy $f = \Delta G / (\lambda \varepsilon_0^2 V)$, after some simple manipulations we get

$$f(x) = \frac{\varphi x^2 (2-x)}{\varphi x^2 (2-x) + AV/r^3} + \frac{\varphi a + (1-\varphi) b x}{\varphi x (2-x)} + \frac{r^3}{V} c \varphi x (2-x), \quad (2)$$

with

$$a = \frac{2\Delta\gamma_*}{\lambda \varepsilon_0^2 r}, \quad b = \frac{2\gamma^*_{GaAs-Ga}}{\lambda \varepsilon_0^2 r}, \quad c = \frac{\Delta\gamma}{\lambda \varepsilon_0^2 r}, \quad (3)$$

$\Delta\gamma_* = \gamma^*_{GaAs-SiO_x} + \gamma^*_{GaAs-Ga} - \gamma^*_{SiO_x-Ga}$ as the vertical and $\Delta\gamma = \gamma_{GaAs-Si} + \gamma_{GaAs-Ga} - \gamma_{Si-Ga}$ as the in-plane surface energy change. When $x << 2$, Eq. (2) is simplified to

$$f(x) = \frac{\varphi x^2}{\varphi x^2 + Av} + \frac{a}{2x} + \frac{c\varphi x}{v}, \quad (4)$$

where we do not write an unimportant x-independent constant. The parameter $v = V/(2r^3) \cong \pi(V/V_{hole})$ is proportional to the ratio of the GaAs volume to the effective total volume of the hole $V_{hole} = 2\pi r^3$ at the hole height $h = 2r$ corresponding to the hole aspect ratio of 1. Clearly, the x dependence of the free energy given by Eq. (4) is controlled by five parameters, the volume coefficient $v << 1$, the elastic energy relaxation A, the opening angle φ, and the two surface energy coefficients a and c describing the changes of the vertical and in-plane surface energies upon nucleation of a GaAs crystal in the hole. The case $c < 0$, $a > 0$ corresponds to the situation where GaAs wets the Si substrate but not the SiO_x walls of the hole [9], consistent with the fact that GaAs crystals grow in two-dimensional (2D) form on silicon and as irregular three-dimensional (3D) crystals on SiO_x [7,12].

The energetically preferred configuration x_0 is now determined by the minimum of $f(x)$ for a given set of parameters. If we assume $va/(2\varphi) << |c|$, the minimum at intermediate x appears due to the elastic energy term in Eq. (4) for sufficiently large GaAs volumes when $Av >> \varphi x^2$ and corresponds to the ring width

$$w_0 \cong \frac{A|\Delta\gamma|}{\lambda\varepsilon_0^2}. \qquad (5)$$

With the known A and $\lambda\varepsilon_0^2$, the experimentally observed ratio $\omega_0 / r \cong 0.25$ for r=30 nm corresponds to a plausible $\Delta\gamma =$-0.225 J/m^2. Suppl. Fig. 9. shows the free energy as a function of x for the full ring geometry of GaAs ($\varphi = \pi$) with these parameters and at $\Delta\gamma_* =$0.05 J/m^2, corresponding to $a/2 =$0.0074 and $c =$ -0.033, for different values of V/V_{hole}.

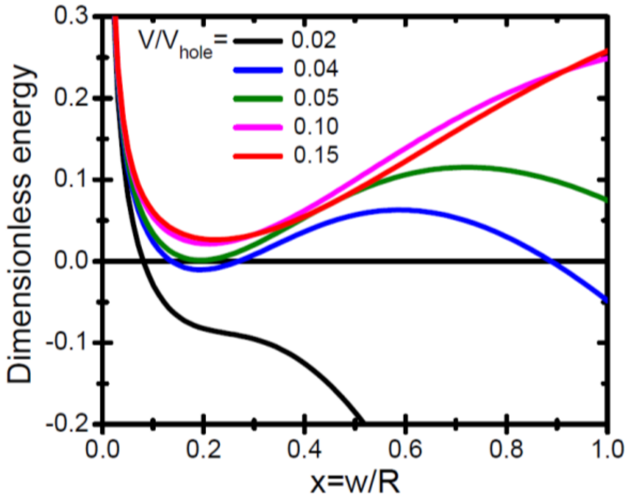

***Supplementary Figure 9**: Free energy versus relative width of full GaAs rings ($\varphi = \pi$) for different V/V_{hole} values, showing the collapse to a volume-independent preferred configuration corresponding to the minimum free energy at $x_0 \cong 0.23$.*

These graphs show the following major properties. For very small GaAs volumes (the curve at $V/V_{hole} = 0.02$), the energetically preferred configuration is 2D GaAs layer. As the GaAs volume increases, the free energy acquires the local minimum at $x \cong$0.2 for the parameters considered, which becomes global at a slightly larger $x_0 \cong$0.23, corresponding to the energetically preferred width $w_0 \cong 0.23R$, as observed in our experiments. Further increase of the GaAs volume does not affect the position of the energy minimum. According to Eq. (5), the width of the ring does not depend on the opening angle, yielding the same width for differently sized gallium droplets in the holes. The height of the rings $h_0 = V/(\varphi R^2 x_0)$ increases linearly with the GaAs volume and becomes larger for smaller φ.

Supplementary Note 7: Arsenic concentration profile in droplet asymmetrically filling the holes and symmetrically filling the holes

The Surface Evolver simulations showed that for small droplet volumes, the equilibrium configuration under cylindrical cavity constrained is the positioning of the droplet at the edges of the cavity. In this part, we analyze the effect of cavity shadowing effect on the Arsenic concentration profile inside the liquid droplet in case of asymmetric hole filling.

The droplet shape is approximated to a cap of a sphere intersecting a cylinder, with the center of the spherical cap in correspondence of the cylindrical sidewalls, as represented in the sketch in Suppl. Fig. 10 a.

In Suppl. Fig. 10 b. the calculated concentration profile is shown in a sliced contour plot along the plane cutting the droplet as depicted in Suppl. Fig. 10 a. The black arrows are drawn to help the reader visualizing the direction of the concentration gradient, which results directed towards the oxide sidewalls and the oxide-substrate contact line.

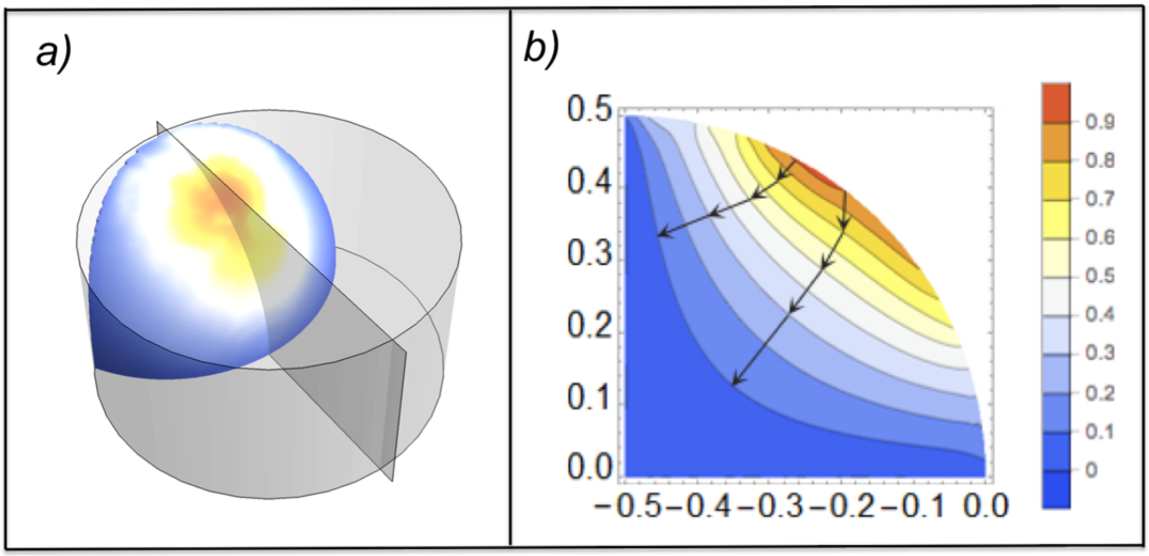

Supplementary Figure 10: *a) Sketch of the droplet position inside a cylindrical cavity used to perform the calculation of the Arsenic concentration profile in the case of asymmetric filling of the holes. b) Sliced contour plot of the calculated Arsenic concentration profile along the plane shown in inset a. The black arrows are drawn to help visualize the direction of the As gradient. The color scale bar is the same for insets a and b.*

For completeness we show in Suppl. Fig. 11. and Fig. 12. the Arsenic concentration profile for the symmetrical filling of the holes, in case of lower and higher filling conditions respectively. The 2 cases differ in the ratio of cavity-sidewalls which results wetted by the liquid. The shadowing effect modifies the inclination of the Arsenic gradient.

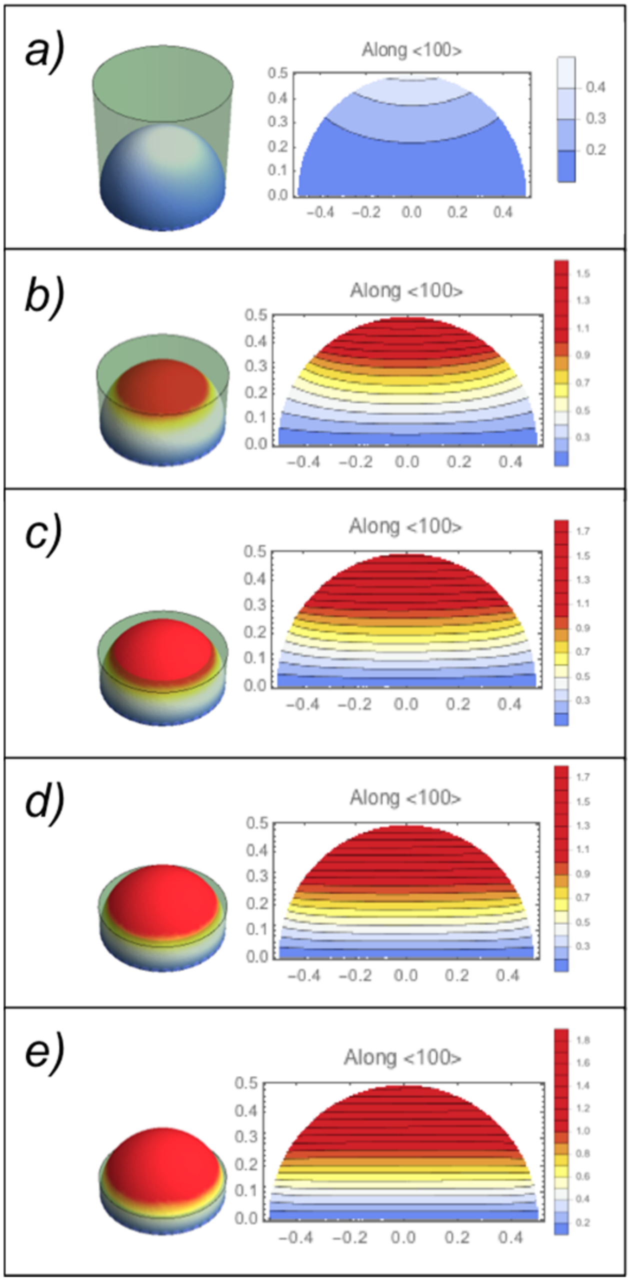

Supplementary Figure 11: *Sketch of the symmetrical low filling droplet configuration inside a cylindrical cavity and corresponded sliced contour plot of the calculated Arsenic concentration profile for different aspect ratio: a) w/h=1, b) w/h=2, c) w/h=3, d) w/h=4 and e) w/h=6.*

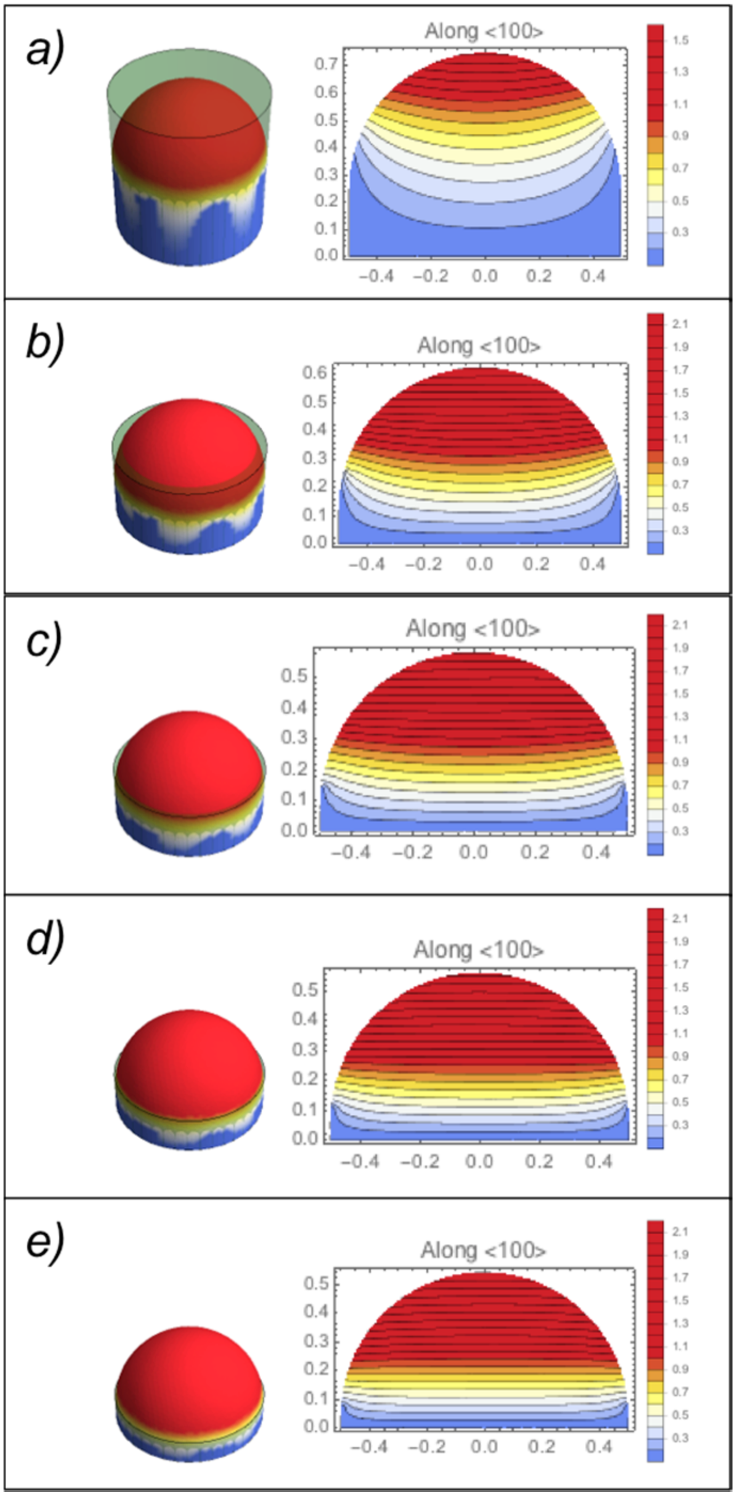

Supplementary Figure 12: *Sketch of the symmetrical high filling droplet configuration inside a cylindrical cavity and corresponded sliced contour plot of the calculated Arsenic concentration profile for different aspect ratio: a) w/h=1, b) w/h=2, c) w/h=3, d) w/h=4 and e) w/h=6.*

In all the cases exposed in this work the calculations were run as follow. The solutions to the Laplacian form of the Fick's law are obtained through Finite Element Method for each orientation of the impinging flux. The final concentration profile of the diffusing material inside the droplet is obtained as a time average of all the solutions calculated in a complete period of rotation of the flux around the z-axis of the cylinder.

The boundary conditions defined to solve the differential equation are maintained the same as for the symmetrical filled holes. We define them here explicitely:

- conc[x, y, z] == 0.1 when z == 0
- conc[x, y, z] == 0.1 when $x^2 + y^2 == (0.5)^2$
- FLUX{conc[x, y, z] } == $\mathrm{ray}[t].\mathrm{Normalize}[\{x, y, z\}]$
  when {x,y,z} $\in$ $\mathrm{illuminatedradius}, t, \mathrm{aspectRatio}]$

The first 2 equations define the absence of diffusion through the substrate and the sidewalls of the cavity. No bouncing or desorption effects were taken into consideration. The last constraint describes the existence of an effective impinging flux just on the surface of the droplet intersecting the time dependent flux. Moreover we defined the flux with an orientation dependent value, that is to say it is maximum when it hits a region of the surface with normal parallel to the direction of the flux itself.

Supplementary Note 8: Fabrication issues on 30 nm hole arrays

In the main text we refer to the lower yield in the aspect ratio holes smaller then 6, particularly the one correspond to 10 nm oxide thickness, assigning it to the fabrication imperfections. These imperfections are manifested like badly defined holes with sizes and shapes differing from nominal one. In addition, for these arrays we find many holes missing; i.e. not being defined at all. All this is exemplified in the Suppl. Fig. 13. In Suppl. Fig. 13a and b we present the comparison of the yields for aspect ratios 5 and 6 (corresponding to 10 nm thick oxide and nominal holes diameters of 30 and 45 nm) for samples grown in the same conditions, for different times (30 min and 5 minutes respectively). For 5 minutes sample it is clearly seen that some holes are missing in the 30 nm arrays while in some holes non-vertical growth occurs. In the case of 45 nm holes this is not the case. In the Suppl. Fig. 13c, we show the SEMs of the substrates before growth. Red arrows point to the places where hole is missing while blue arrows point to the holes that are smaller in diameter or their shape differs from other holes in the array. Again, in 45 nm arrays this is not the case.

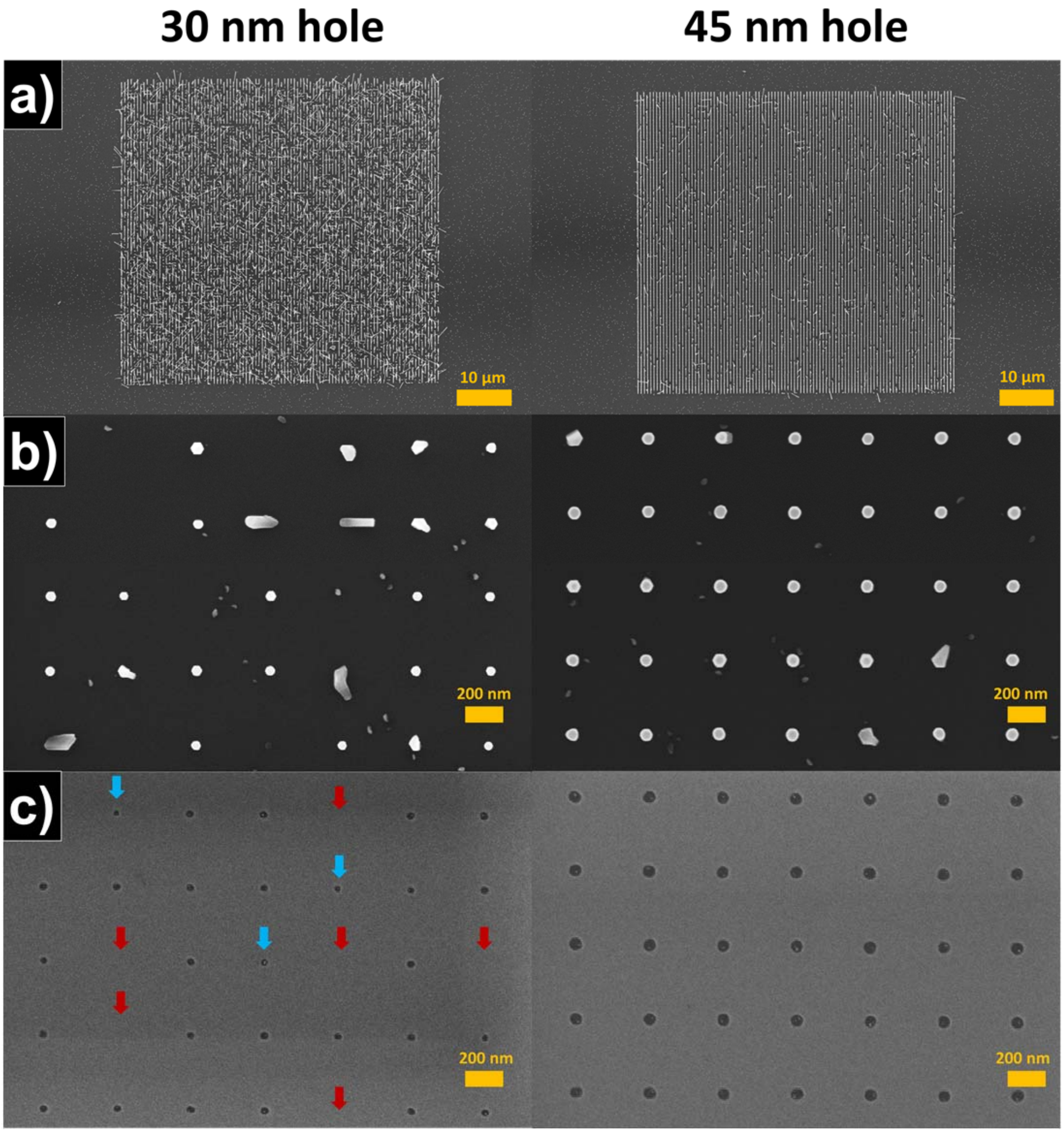

Supplementary Figure 13: *SEM images of NW arrays from 30 nm and 45 nm hole (a) 30 min of growth, (b) 5 min growth and (c) bare hole arrays. (Red arrows indicate the positions where holes are not defined and blue arrows indicate irregularity of holes.)*

References

[1] V. G. Dubrovskii, N. V. Sibirev, J. C. Harmand, and F. Glas, Growth kinetics and crystal structure of semiconductor nanowires. Phys. Rev. B **78**, 235301 (2008).

[2] D. Kashchiev, Dependence of the growth rate of nanowires on the nanowire diameter. Cryst. Growth Des. **6**, 1154 (2006).

[3] V. G. Dubrovskii, Theory of VLS growth of compound semiconductors. In: A. Fontcuberta i Morral, S. A. Dayeh and C. Jagadish, editors, Semiconductors and Semimetals, v. 93, Burlington: Academic Press, 2015, pp. 1-78.

[4] C. Y. Wen, J. Tersoff, K. Hillerich, M. C. Reuter, J. H. Park, S. Kodambaka, E. A. Stach, and F. M. Ross, Periodically changing morphology of the growth interface in Si, Ge, and GaP nanowires, Phys. Rev. Lett. **107**, 025503 (2011).

[5] D. Jacobsson, F. Panciera, J. Tersoff, M. C. Reuter, S. Lehmann, S. Hofmann, K. A. Dick, F. M. Ross, Interface dynamics and crystal phase switching in GaAs nanowires. Nature **531**, 317 (2016).

[6] M. T. Robson, V. G. Dubrovskii, and R. R. LaPierre, Conditions for high yield of selective-area epitaxy InAs nanowires on SiOx/Si(111) substrates. Nanotechnology **26**, 465301 (2015).

[7] J. Vukajlovic-Plestina, V. G. Dubrovskii, G. Tütüncuoğlu, H. Potts, R. Ricca, F. Meyer, F. Matteini, J.-B. Leran, and A. Fontcuberta i Morral, Molecular beam epitaxy of InAs nanowires in SiO_2 nanotube templates: challenges and prospects for integration of III–Vs on Si. Nanotechnology **27**, 455601 (2016).

[8] F. Glas, Critical dimensions for the plastic relaxation of strained axial heterostructures in free-standing nanowires, Phys. Rev. B **74**, 121302 (R) (2006).

[9] V. G. Dubrovskii, N. V. Sibirev, X. Zhang, and R. A. Suris, Stress-driven nucleation of three-dimensional crystal islands: from quantum dots to nanoneedles. Cryst. Growth Des. **10**, 3949 (2010).

[10] X. Zhang, V. G. Dubrovskii, N. V. Sibirev, and X. Ren, Analytical study of elastic relaxation and plastic deformation in nanostructures on lattice mismatched substrates. Cryst. Growth Des. **11**, 5441 (2011).

[11] G. E. Cirlin, V. G. Dubrovskii, I. P. Soshnikov, N. V. Sibirev, Yu. B. Samsonenko, A. D. Bouravleuv, J. C. Harmand, and F. Glas, Critical diameters and temperature domains for MBE growth of III-V nanowires on lattice mismatched substrates. Phys. Stat. Sol. RRL **3**, 112 (2009).

[12] J. Vukajlovic-Plestina, W. Kim, V. G. Dubrovskii, G. Tütüncüoğlu, M. Lagier, H. Potts, M. Friedl, and A. Fontcuberta i Morral, Engineering the size distributions of ordered GaAs nanowires on silicon. Nano Lett. **17**, 4101 (2017).
